# Synergistic effects of levo-tetrahydropalmatine and low-dose naltrexone on nicotine conditioned place preference in mice: a dual-target strategy based on dopamine and opioid systems

**DOI:** 10.3389/fphar.2026.1697258

**Published:** 2026-01-14

**Authors:** Kun Feng, Yiran Zhao, Lu Liu, Shan Kang, Mingming Yu, Sherwin K. B. Sy, Zhihua Lv, Meixing Yan

**Affiliations:** 1 School of Medicine and Pharmacy, Ocean University of China, Qingdao, China; 2 Women and Children Hospital, Qingdao University, Qingdao, China; 3 Department of Statistics, State University of Maringá, Maringá, Paraná, Brazil

**Keywords:** combination therapy, conditioned place preference model, levo-tetrahydropalmatine, low-dose naltrexone, nicotine addiction

## Abstract

**Background:**

Nicotine addiction is a major public health challenge, with existing pharmacological interventions often limited by suboptimal efficacy, adverse effects and withdrawal symptoms.

**Objectives:**

This study explores the effects of combining levo-tetrahydropalmatine (l-THP), a dopamine receptor antagonist, with low-dose naltrexone (LDN), an opioid receptor antagonist, on nicotine-induced conditioned place preference (CPP) in mice.

**Methods:**

The combined therapeutic effects of l-THP and LDN on nicotine was evaluated using a mouse CPP paradigm. Male Kunming mice were subjected to nicotine-induced CPP, followed by treatment with l-THP, LDN, or their combination. Behavioral assessments were conducted, and plasma β-endorphin levels were measured using enzyme linked immunosorbent assay.

**Results:**

A 10 mg/kg l-THP alone significantly attenuated CPP, while LDN alone showed no significant effect. The combination of 10 mg/kg l-THP and 0.3 mg/kg LDN produced a synergistic reduction in nicotine-seeking behavior, effectively reversing the CPP effect. The combination therapy was associated with an increased plasma β-endorphin levels, suggesting a modulation of the endogenous opioid system.

**Conclusion:**

These findings indicate that the combination therapy based on dual-action mechanism of l-THP and LDN, targeting both dopamine and opioid pathways, effectively attenuates nicotine-induced CPP in mice, which may offer a potential treatment for nicotine addiction. The combination enhances therapeutic efficacy within the nicotine-induced CPP paradigm and correlates with an elevation of β-endorphin levels.

## Graphical Abstract

**Figure:**
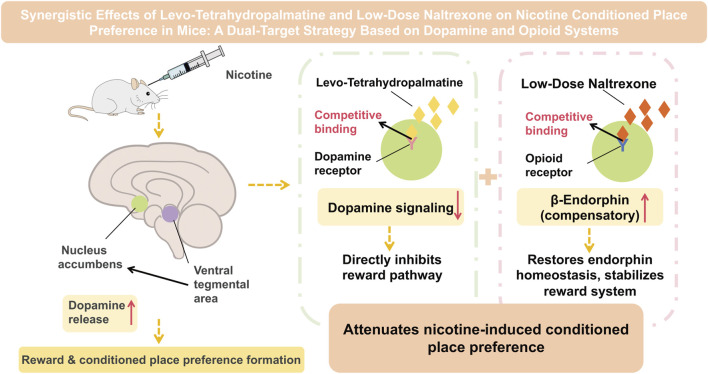

## 1 Introduction

The World Health Organization (WHO) reported that there were over eight million deaths annually worldwide due to smoking-related diseases (Samarasekera, 2023). However, only a fraction of smokers managed to quit due to their high addiction to nicotine. Nicotine affects people’s emotions through the mesolimbic dopamine reward pathway (Di Chiara and Bassareo, 2007; Hollander et al., 2008) and the synergy between many other key brain neurotransmitters, including serotonin (5-HT), glutamate (Glu), and gamma-aminobutyric acid (GABA) (Commons, 2008; Sperling and Commons, 2011).

Varenicline and bupropion are the most widely prescribed medicines to manage nicotine addiction (Rahimi et al., 2024; Yan and Goldman, 2021; Zierler-Brown and Kyle, 2007). Varenicline, a partial nicotinic acetylcholine receptor (nAChR) agonist, reduces nicotine-induced rewarding effects but is associated with significant adverse reactions, including nausea, dizziness, and neuropsychiatric complications (Coe et al., 2005; Prochaska and Benowitz, 2019; Rollema et al., 2007). Although bupropion, a norepinephrine-dopamine reuptake inhibitor (NDRI), could increase dopamine levels simultaneously, the side effects of anxiety and insomnia made it an impediment to its wide application (Cryan et al., 2003; Hurt et al., 1997). Current treatment strategies have achieved certain progress in promoting nicotine cessation, but they are still limited by suboptimal therapeutic efficacy, significant adverse effects, and a high rate of re-addiction (Benowitz, 2009). Due to the complexity of nicotine addiction mechanisms, drugs targeting other relevant pathways may offer additional therapeutic benefits (Benowitz, 2010).

Levo-tetrahydropalmatine (l-THP) is an alkaloid extracted from the herb *Corydalis yanhusuo* (Liu et al., 2012). Many studies showed that l-THP primarily exerted its effects through the antagonism of D1, D2 and D3 dopamine receptors (Liu et al., 2019). The D3 receptor had been identified as an important target to prevent relapse in addiction treatment (Xi et al., 2004). L-THP significantly reduced nicotine self-administration and attenuated nicotine-seeking behavior following re-exposure, indicating that it has the potential as an alternative for varenicline and bupropion (Faison et al., 2016). The effects of l-THP in reducing the rewarding effects induced by addictive substances such as cocaine, oxycodone, and heroin had also been investigated, which further supported its role in addiction treatment. However, the dose-dependent sedative effect of l-THP limited its standalone use in addiction treatment (Xi et al., 2007; Xu et al., 2013).

Naltrexone, a competitive antagonist of opioid receptors, is used for the treatment of alcohol and opioid dependencies (Resnick et al., 1974; Wang et al., 2007), by blocking the rewarding effects of addictive substances (Modesto-Lowe and Van Kirk, 2002). At conventional therapeutic doses of 50–250 mg, naltrexone reduces cocaine-seeking behavior in rodents (Schmitz et al., 2009; Schmitz et al., 2001). In clinical trials testing standard therapeutic dose of 50 mg per day naltrexone, there were significant adverse reactions, poor tolerance, and high dropout rates, resulting in suboptimal clinical efficacy (Grassi et al., 2007; Schmitz et al., 2014; Schmitz et al., 2009). In contrast, studies using low-dose naltrexone (LDN), defined as a daily dose of <5 mg, demonstrated improvements in alleviating withdrawal symptoms (Rahn et al., 2011; Zagon et al., 2014). LDN prevailed over high dose of naltrexone in its anti-addictive effects (Brown and Panksepp, 2009).

Recently, it was shown that the combination of LDN and l-THP significantly attenuated cocaine-induced addiction and relapse compared to l-THP alone (Sushchyk et al., 2016). Since the mechanisms of nicotine addiction were similar to cocaine addiction, the combination of LDN and l-THP was proposed as a potential therapeutic approach through targeting both dopamine and opioid receptors. LDN may mitigate the sedative effects induced by l-THP through modulation of the endogenous opioid system. The combination therapy could reduce the required therapeutic doses of each drug and minimize their side effects. In this study, we evaluated the effects of this combination therapy on nicotine-induced conditioned place preference (CPP) in mice, which is commonly used as a model of nicotine reward learning.

## 2 Materials and methods

### 2.1 Animals

Male Kunming strain mice (SPF grade), weighing 25–30 g, were purchased from Jinan Pengyue Experimental Animal Breeding Co., Ltd. (Animal Production License No. SCXK-20190003). The animals were housed in an environment with a temperature of 25 ± 2 °C, relative humidity of 50–55%, and were provided *ad libitum* access to food and water. Before the experiment, a 1-week acclimatization period was performed, including a 12-h fasting period before intraperitoneal injections. All animal experimental protocol were approved and conducted in compliance with the guidelines set by the Animal Ethics Committee of the School of Medicine and Pharmacy, Ocean University of China (Approval No. OUC-SMP-2019–10–10).

### 2.2 Nicotine and drug preparation

*Nicotine Injection*: (−)-Nicotine (S, free base) was dissolved in 0.9% saline, and the pH was adjusted to 7 ± 0.2 using sodium hydroxide. The final concentration was 0.05 mg/mL. *L-THP Injection*: l-THP was dissolved in 0.1 M H_2_SO_4_, and the pH was adjusted to 5.5 ± 0.2 using 0.1 M NaOH. The volume was adjusted with sterile water to a final concentration of 1.0 mg/mL. *Naltrexone Injection*: Naltrexone powder was accurately weighed and dissolved in 0.9% saline to a final concentration of 0.03 mg/mL. *L-THP and Low-Dose Naltrexone (LDN) Mixture Injection*: Naltrexone was dissolved in the prepared l-THP solution for immediate use. The solution was sterilized using high-temperature, high-pressure autoclaving and stored in amber reagent bottles sealed with foil at 4 °C. All solutions were used within 7 days from the time they were prepared. Before each use, the injection solution was filtered through a 0.22-µ filter to prevent contamination.

### 2.3 Apparatus

The place conditioning paradigm model, characterized by a short experimental duration and simple methodology, had been extensively used in drug reward research. The time spent by mice in the drug-paired chamber (which was the less-preferred white chamber in our biased paradigm) was used to assess the degree of drug seeking, making it a classic animal model for screening anti-addiction medications (Asth et al., 2022; Katz and Gormezano, 1979).

The CPP experiments were conducted using the XR-XT401 CPP System (Shanghai Xinruan), which employed computer-based video tracking and communication technology. The system comprised of hardware (CPP box, gun-type camera, bracket, and capture card) and software (SuperMaze). The CPP box consisted of two compartments (40 cm × 40 cm × 40 cm each), one black compartment with smooth walls and a textured floor with circular holes, and the other white compartment with soft walls and a textured floor with stripes. The compartments were separated by a removable partition, allowing free movement between them when the partition was removed.

### 2.4 Nicotine-induced CPP model in mice

In this experiment, there were several factors examined for establishing the nicotine-induced CPP in mice, including the type of animal, acclimatization training time, nicotine administration method during training, nicotine dosage, training duration, and duration of each session. The procedure consisted of four stages: (1) acclimatization stage (1 day), (2) baseline testing stage (2 days), (3) training stage (5 days), and (4) testing stage (1 day). The conditions were optimized before running the CPP model, as listed in Supplementary Table S1.

During the acclimatization stage, mice were transferred to an experimental room and were allowed to freely explore the cage for an hour. The mice were then gently handled by rubbing the skin on their necks to minimize fear reactions during subcutaneous injection. Afterward, the mice were individually placed at the center of the CPP chamber, and the partition was removed to allow mice to move freely for 15 min to acclimatize to the environment. There was no data recorded during this stage.

The next step was to test the baseline value. First, the skin on the neck of mice was gently rubbed before the mice were placed at the center of the CPP chamber. After that, the mice were allowed to move freely for 15 min; simultaneously, their time spent in the white and black chambers was recorded. The chamber where the mice spent less time was assigned as the drug-paired chamber. Because 90% of the mice naturally preferred the black compartment, a biased place conditioning paradigm was used. Nicotine was paired with the initial least preferred compartment (the white chamber), while the black compartment was used for saline injection. This baseline test was performed over 2 days, and the average of the two test results was defined as the baseline time spent in the drug-paired chamber. The results from mice with a strong innate preference (defined as spending more than 70% of the session in either compartment) were excluded to eliminate potential biases.

As for the training stage, all mice were divided into the nicotine experimental group and the saline control group. There were two training sessions conducted daily, with the partition separating the two chambers intact so that the mice could only move in one chamber. Locomotor activity was also simultaneously recorded. Both the total distance traveled (mm) and mean velocity (mm/s) were extracted as parameters to ensure that the observed CPP effects were not confounded by changes in their general motor activity.

The testing stage was carried out for 2 days after the completion of their training (24 h after the last injection). The partition was removed to allow mice to move freely between the black and white chambers for 15 min; the time spent in the white chamber was recorded. The CPP score 1 was calculated by subtracting the baseline time spent in the white chamber from the time spent there during the testing stage, which was used to assess the reward effect of nicotine and determine whether the nicotine-induced CPP model had been successfully established.

### 2.5 Treatment group and training scheme

After confirming a successful establishment of nicotine-induced CPP, mice were rank-ordered according to their CPP score 1 value and assigned to treatment groups using block randomization, ensuring comparable baseline CPP scores across groups. Each treatment group consisted of nine mice. The groups were set as follows: 1) saline control, 2) nicotine control, 3) 0.3 mg/kg LDN, 4) 5 mg/kg L-THP, 5) 10 mg/kg L-THP, 6) 0.3 mg/kg LDN +5 mg/kg L-THP, and 7) 0.3 mg/kg LDN +10 mg/kg L-THP. The training protocol for each group is detailed in Table 1.

**TABLE 1:** Experimental design and training scheme for drug testing using the conditioned place preference model.

Group	White box		Black box	
	Pre-training 30 min	Training	Pre-training 30 min	Training
1	Saline	Saline	Saline	Saline
2	Saline	0.5 mg/kg nicotine	Saline	Saline
3	0.3 mg/kg LDN	0.5 mg/kg nicotine	Saline	Saline
4	5 mg/kg l-THP	0.5 mg/kg nicotine	Saline	Saline
5	10 mg/kg l-THP	0.5 mg/kg nicotine	Saline	Saline
6	0.3 mg/kg LDN+ 5 mg/kg l-THP	0.5 mg/kg nicotine	Saline	Saline
7	0.3 mg/kg LDN+ 10 mg/kg l-THP	0.5 mg/kg nicotine	Saline	Saline

l-THP: levo-tetrahydropalmatine; LDN: low-dose naltrexone.

### 2.6 Experimental design

In both monotherapy and combination therapy experiments, mice with successfully established nicotine-induced CPP models were used. After nicotine CPP was successfully established, mice continued to undergo additional nicotine conditioning sessions. Pharmacological interventions (l-THP, LDN, or their combination) were administered 30 min before each nicotine injection. This design allowed us to evaluate whether drug intervention could attenuate the maintenance or expression of the established nicotine CPP. Nicotine training and drug administration were conducted simultaneously, and mice were treated with the respective drug and dose 30 min before each administration of nicotine as outlined in Table 1. After 5 days, the changes in time spent in the white box were calculated using CPP score 2 (CPP score 2 = time spent in the white box after drug treatment - time spent in the white box before drug treatment). A schematic timeline summarizing cohorts and CPP scoring across all experimental phases is provided in Supplementary Figure S1.

### 2.7 Blood plasma β-endorphin measurement

After mice completed their nicotine CPP training and their CPP scores recorded, their blood samples were collected within 30 min post-training. Mice from the nicotine control, 0.3 mg/kg LDN, 10 mg/kg l-THP, and 0.3 mg/kg LDN +10 mg/kg l-THP groups (8 mice per group) were anesthetized, and blood samples were collected into 2 mL centrifuge tubes containing ethylenediaminetetraacetic acid (EDTA) anticoagulant plus antiprotease and then centrifuged at 4,000 *g* for 15 min at 4 °C. Plasma was separated, labeled, sealed, and stored at −80 °C until analysis. The β-endorphin levels in the plasma were measured using enzyme linked immunosorbent assay (ELISA). Correlation analyses were conducted between behavioral measures (CPP score 2) and biochemical measures (β-endorphin plasma concentrations). For normally distributed datasets, Pearson correlation coefficient was calculated; Spearman’s rank correlation was used when the normality assumption was not met.

### 2.8 Statistical analysis

Data were plotted using GraphPad Prism 10 (GraphPad Software, San Diego, CA, USA), and results are presented as mean ± SEM. Statistical analyses were performed using R version 4.4.1 (R Foundation, Vienna, Austria) or GraphPad Prism 10. Statistical significance was defined as p < 0.05.

For comparisons between two independent groups, an unpaired two-tailed Student’s t-test was used. For comparisons among three or more independent groups, data were compared using one-way analysis of variance (ANOVA) implemented within a linear model algorithm. Levene’s test and Shapiro-Wilk test was applied to assess homogeneity of variances and normality of residuals, respectively. When the overall ANOVA was significant, Tukey’s honestly significant difference (HSD) test was used for *post hoc* pairwise comparisons to control the family-wise type I error rate. When the variance homogeneity and normality assumptions were violated, the Kruskal–Wallis test followed by Dunn’s *post hoc* test was applied.

For analyses comparing different conditioning stages within the same treatment group (baseline, nicotine-training, and drug training), data were tested for normality using the Shapiro-Wilk test. For datasets meeting normality assumptions, one-way repeated measures ANOVA followed by Tukey’s HSD was used. When normality was violated, the Friedman test with Dunn’s *post hoc* correction was applied.

The association between two continuous variables was examined using Pearson’s correlation coefficient.

Sample size estimation was performed based on preliminary data obtained from pilot experiments. Assuming between-group mean differences ranging from 80 to 95 s and a standard deviation of 65 s, a power analysis using the t-statistics for independent samples (α = 0.05, two-tailed; power = 80%) indicated that approximately 8–11 animals per group would be required to detect such effects.

## 3 Experimental results

### 3.1 Evaluation of factors affecting CPP model establishment

#### 3.1.1 Animal species selection

Two types of laboratory mice were considered for this experiment, namely, Kunming and C57BL/6 mice. Under consistent experimental conditions tested, Kunming mice were determined to be more docile, exhibiting less resistance to experimental procedures, and showing milder stimulation responses to nicotine injections compared to C57BL/6 mice. Consequently, Kunming mice were chosen for establishing the nicotine-induced CPP model.

#### 3.1.2 Adaptation training

A 1-day adaptation session was used to familiarize mice with the CPP apparatus and minimize baseline variability. Mice that underwent this adaptation exhibited more stable initial preference patterns compared with mice without prior training. Extending the adaptation beyond 1 day did not further improve baseline stability and instead reduced animal activity, which could compromise subsequent conditioning results. Therefore, a single day of adaptation was selected as the optimal procedure for all experiments.

#### 3.1.3 Intraperitoneal vs. subcutaneous nicotine injection and dosage effects on establishing the CPP model

The following intraperitoneal doses were used for nicotine training: 0.5, 1.0, and 2.0 mg/kg (Supplementary Figure S2). A significant dose effect on CPP following intraperitoneal nicotine administration was observed (ANOVA F (3, 28) = 6.30, p = 0.0021). In the 0.5 mg/kg and 1.0 mg/kg groups, mice spent more time in the white box compared to the saline control group. The 1.0 mg/kg dose reliably induced a significant CPP (*post hoc* Tukey’s test, P < 0.05). In contrast, 2.0 mg/kg nicotine failed to produce a CPP and instead tended to induce an aversive response, suggesting that the higher dose may have produced dysphoric effects that interfered with CPP acquisition.

The subcutaneous injection group was assigned three dosage levels: 0.25, 0.5, and 1.0 mg/kg (Supplementary Figure S3). A significant dose-dependent effect was observed (ANOVA F (3, 28) = 5.10, p = 0.0061). Among the three doses tested, only the 0.5 mg/kg dose reliably induced a significant CPP (*post hoc* Tukey’s test, P < 0.01). Therefore, subcutaneous injection of 0.5 mg/kg nicotine was selected as the optimal administration method for establishing the nicotine-dependent CPP model.

#### 3.1.4 Training duration

The most robust nicotine-induced place preference was established in mice that undergone 5 days of training with nicotine subcutaneous administration for 30 min per session. CPP was not established in mice with 3 days or 7 days of training due to insufficiency and aversion, respectively.

#### 3.1.5 Optimization of effective establishment process to the CPP model

**FIGURE 1:**
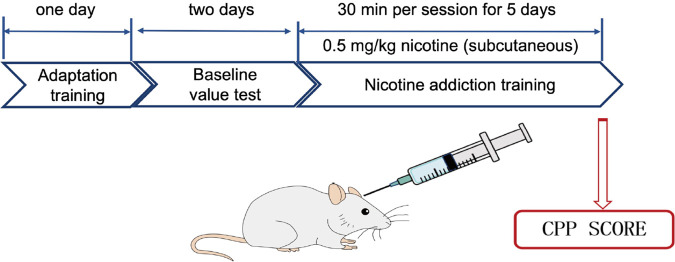
Illustrated experimental design of nicotine-induced conditioned place preference. Kunming mice were given 1 day of adaptation training. During nicotine conditioning, mice received subcutaneous injections of nicotine at 0.5 mg/kg, with each conditioning session lasting 30 min, and the training was conducted once daily for five consecutive days.

The standardized protocol for establishing a reliable nicotine-induced CPP model was developed (Figure 1). The following standardized conditions were set-up for an optimal CPP model: (1) Kunming mice were selected due to their consistent behavioral responses; (2) a 24-h acclimatization period was implemented to minimize environmental stress and ensure behavioral baseline stability; (3) each nicotine conditioning session was maintained at 30 min to achieve optimal associative learning while preventing fatigue; (4) the training protocol spanned five consecutive days to ensure robust CPP formation, consisting of alternating nicotine-paired and saline-paired sessions; (5) nicotine administration was performed through subcutaneous injection at the dorsal neck region to ensure consistent pharmacokinetics; and (6) the optimal nicotine dosage was determined to be 0.5 mg/kg, which effectively induced CPP. This standardized protocol had been validated to produce consistent and reproducible CPP results, providing a reliable platform for subsequent experiments to evaluate effectiveness of drug intervention.

#### 3.1.6 Qualification of nicotine-induced CPP

**FIGURE 2:**
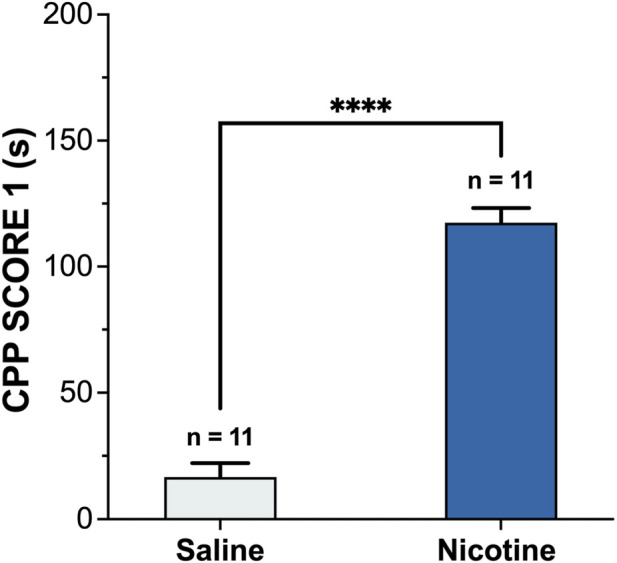
Effect of nicotine on conditioned place preference (n = 11 per group, Mean ± SEM). The animal received 0.5 mg/kg subcutaneous nicotine for 5 days. CPP score 1 reflects the nicotine-induced change in white compartment preference (calculated as post-nicotine training time minus pre-test time). Statistical analyses were performed using two-tailed Student’s t-test. ****P < 0.0001.

**FIGURE 3:**
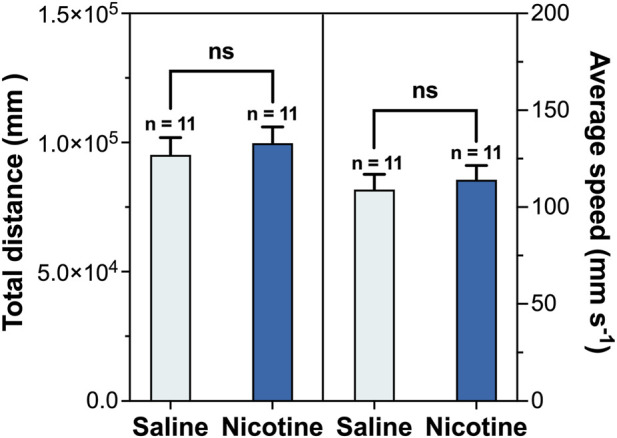
Locomotor activity during the conditioned place preference evaluation (n = 11 per group, Mean ± SEM). Total distance traveled (left Y-axis) and mean velocity (right Y-axis) were measured in saline and nicotine treated groups. Statistical analyses were performed using two-tailed Student’s t-test. No significant differences were observed between groups (P > 0.05).

The saline control group (Figure 2) showed no significant change in time spent in the white box. In contrast, mice receiving nicotine developed a robust and significant CPP, demonstrating a clear shift toward the nicotine-paired environment (statistical significance determined by an unpaired two-tailed Student’s t-test, P < 0.0001). Collectively, these results demonstrated that nicotine could induce a significant place preference effect, and the nicotine-induced CPP model was successfully established. To exclude the possibility that the observed CPP effects were confounded by nonspecific motor alterations, locomotor activity during the initial CPP test was recorded and analyzed. No significant differences in total distance traveled or mean velocity were observed among groups during the conditioning sessions (Figure 3), indicating that nicotine treatment did not alter general locomotor activity throughout CPP acquisition.

### 3.2 Effect of l-THP and LDN monotherapy on nicotine-induced CPP in mice

Mice with established nicotine CPP were treated with l-THP or LDN to assess treatment efficacy. The nicotine control group maintained a strong CPP, demonstrating persistence of the nicotine-associated preference in the absence of treatment. A significant effect of treatment on nicotine-induced CPP was observed (ANOVA F (6, 56) = 13.60, p < 0.0001). LDN (0.3 mg/kg) did not significantly alter the established CPP (evaluated by *post hoc* Tukey’s tests), suggesting minimal efficacy when administered alone at this concentration. Treatment with 5 mg/kg l-THP produced only a mild reduction in nicotine-induced CPP that did not reach statistical significance. In contrast, 10 mg/kg l-THP markedly attenuated the nicotine-induced CPP, indicating a dose-dependent reduction in nicotine-associated contextual preference (*post hoc* Tukey’s tests, P < 0.05).

### 3.3 Effect of l-THP and LDN combination therapy on nicotine-induced CPP in mice

**FIGURE 4:**
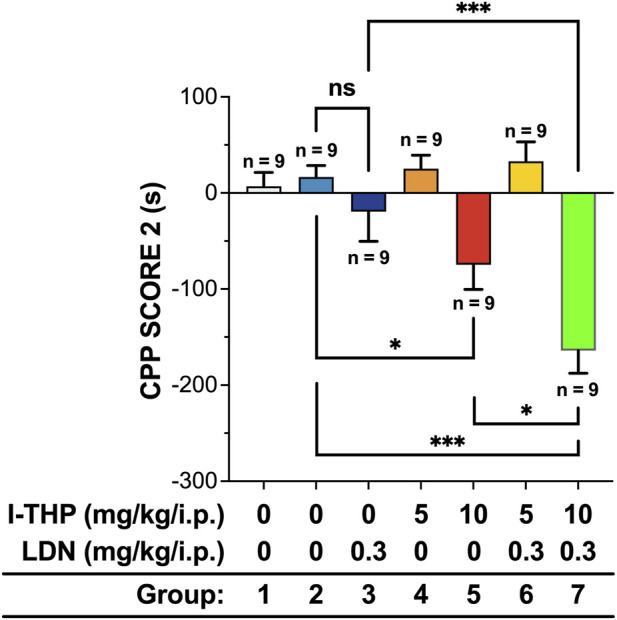
Dose-effect relationship of l-THP, LDN and combined administration on conditioned place preference (n = 9 per group, Mean ± SEM). CPP score 2 indicates the mean change in preference after the administration of l-THP and LDN (post-drug training time minus pre-drug training time). Statistical analyses were performed using one-way analysis of variance followed by *post hoc* Tukey’s HSD test. *P < 0.05, ***P < 0.001.

Two groups of mice (n = 9 per group) were used to examine the effects of combined l-THP and LDN treatment on nicotine-induced CPP: 0.3 mg/kg LDN + 5 mg/kg l-THP, and 0.3 mg/kg LDN + 10 mg/kg l-THP. As shown in Figure 4, a significant effect of treatment on nicotine-induced CPP was observed (ANOVA F (6, 56) = 13.60, p < 0.0001). The high-dose combination (0.3 mg/kg LDN +10 mg/kg l-THP) produced the largest reduction in CPP, showing significantly greater attenuation than either monotherapy (p < 0.05 vs. 10 mg/kg l-THP; p < 0.001 vs. 0.3 mg/kg LDN by *post hoc* Tukey’s tests), as well as a significantly lower CPP than the nicotine control group (p < 0.001). In contrast, the low-dose combination (0.3 mg/kg LDN + 5 mg/kg l-THP) did not significantly alter CPP relative to the nicotine control group, consistent with the lack of effect observed for each low-dose monotherapy. These findings indicate that the combination of high-dose l-THP and LDN produces the most pronounced suppression of nicotine-induced CPP among all groups, demonstrating a clear synergistic therapeutic effect. Although l-THP and l-THP/LDN treatments reduced the time spent in the nicotine-paired compartment relative to the post-nicotine stage, these values consistently remained above baseline levels.

**FIGURE 5:**
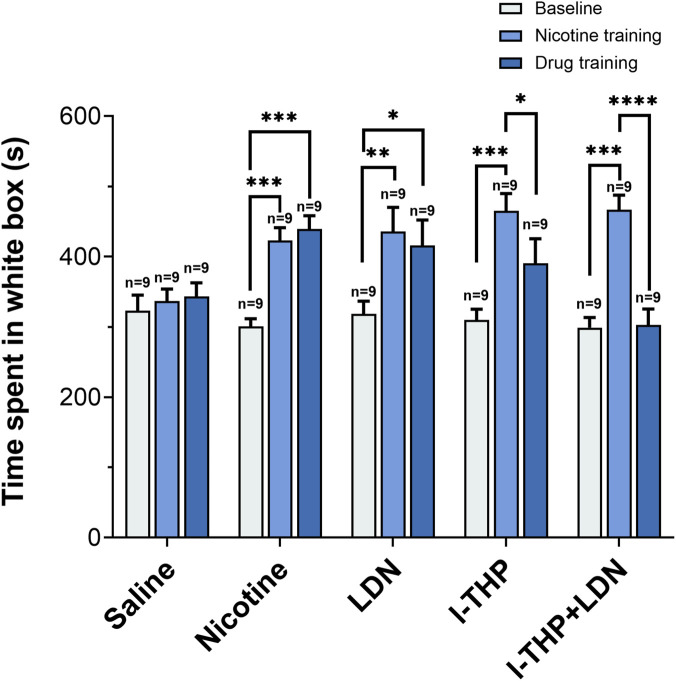
Changes in the time mice spent in the white box (n = 9 per group, Mean ± SEM). Baseline: the initial measurement before any training. Nicotine training: test value after 5 days of nicotine training (Saline control received equivalent volume of saline instead of nicotine training). Drug training: test value after 5 days of drug administration. All groups received a 5-day training. Statistical analyses were performed using one-way repeated-measures analysis of variance followed by *post hoc* Tukey’s HSD test. *P < 0.05, **P < 0.01, ***P < 0.001, ****P < 0.0001.

To evaluate whether pharmacological interventions elicited conditioned aversion, we examined changes in the time spent in the white compartment across baseline, post-nicotine conditioning, and post-treatment stages (Figure 5). After nicotine conditioning, the time spent in the white compartment increased. Following pharmacological intervention, the time spent in the white compartment decreased compared with the post-nicotine stage in the l-THP and LDN/l-THP combination groups, while the values remained above baseline levels.

### 3.4 Measurement of plasma β-endorphin levels

**FIGURE 6:**
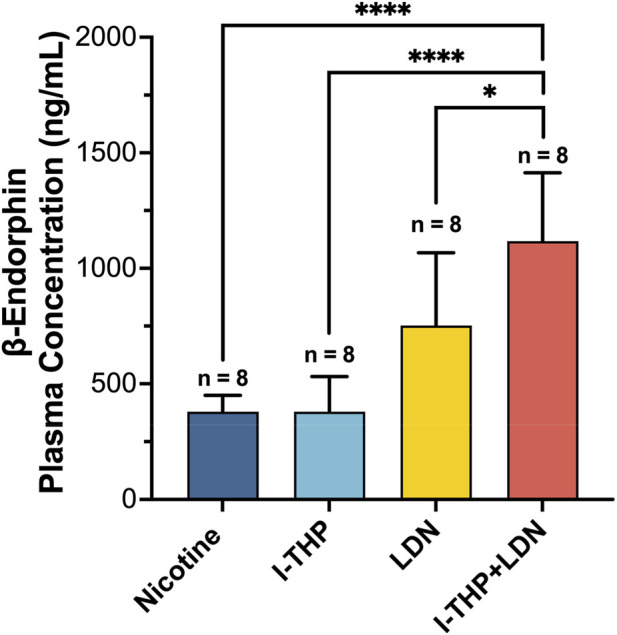
Concentration of plasma β-endorphin (n = 8 per group, Mean ± SEM). Statistical analyses were performed using one-way analysis of variance followed by *post hoc* Tukey’s HSD test. *P < 0.05, ****P < 0.0001.

As shown in Figure 6, plasma samples were collected within 30 min after the completion of the place preference test to measure β-endorphin levels. A significant effect of treatment on plasma β-endorphin concentrations was observed (ANOVA F (3, 28) = 18.55, p < 0.0001). Post hoc Tukey’s tests showed that the combination of 10 mg/kg l-THP and 0.3 mg/kg LDN significantly increased plasma β-endorphin levels compared to the nicotine control group (P < 0.0001), 10 mg/kg l-THP group (P < 0.0001) and 0.3 mg/kg LDN group (P < 0.05).

### 3.5 Correlation analysis between behavioral and biochemical parameter

**FIGURE 7:**
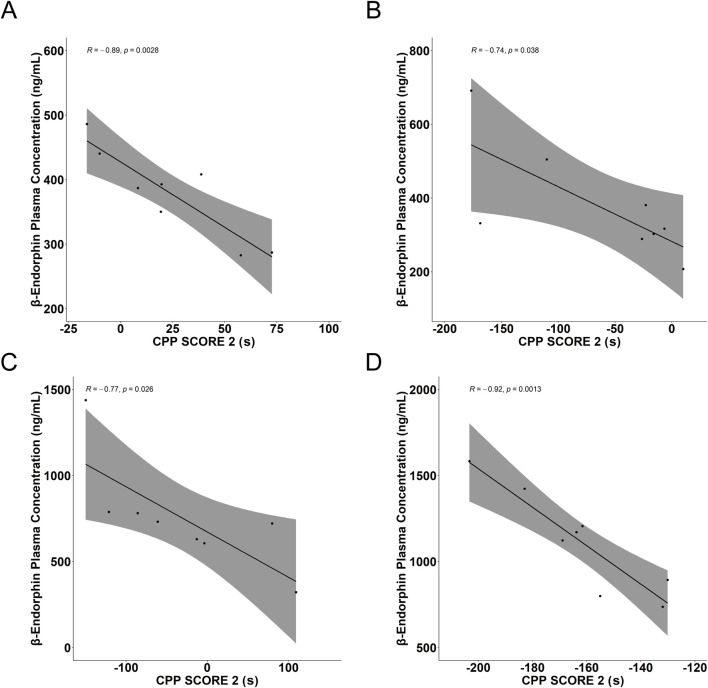
Correlation between β-endorphin plasma concentration and CPP score 2. Correlations were assessed using Pearson’s correlation coefficient. **(A)** Nicotine group (R = −0.89, P = 0.0028), **(B)** LDN group (R = −0.74, P = 0.038), **(C)** l-THP group (R = −0.77, P = 0.026), **(D)** l-THP + LDN group (R = −0.92, P = 0.0013). The shaded area represents the 95% confidence interval of the regression line.

To further examine the relationship between behavioral and biochemical changes, a correlation analysis was performed between CPP score 2 and β-endorphin plasma concentrations across all treatment groups. As illustrated in Figure 7, β-endorphin levels were negatively correlated with CPP score 2 (R = −0.92 to −0.74, p < 0.05).

## 4 Discussion and conclusion

Substance abuse disorders are difficult to treat if targeting only a single pathway because complex mechanisms are involved in drug addiction (Sushchyk et al., 2016). For the same reason, combination therapy has been increasingly utilized in clinical practice, particularly for the treatment of psychiatric disorders (Bailey and Husbands, 2014). Nicotine addiction, which represents a form of psychiatric disorders induced by nicotine abuse, exhibits an identical dilemma with other types of mental disorders. Several pharmacological agents, such as bupropion, varenicline, and nicotine replacement therapy, are used to treat nicotine addiction. But the effectiveness of these treatments for smoking cessation ranged from 5% to 35% only (Benowitz, 2009).

In this study, the effects of l-THP, LDN, and their combination on nicotine-induced CPP were evaluated by recording the time mice spent in the nicotine-paired compartment, as a measure of nicotine reward learning. Locomotor activity during the CPP test was also analyzed, and no significant difference in total distance traveled or mean velocity were observed among groups, indicating that the CPP effects were independent of general motor activity. Mice which had received subcutaneous injection of 0.5 mg/kg nicotine for five consecutive days significantly increased their time spent in the white chamber compared to the baseline value during the pre-test phase, which contradicted their inherent preference for the black compartment. Nicotine, as a rewarding substance of abuse, provided a conditioned stimulus and induced a conditioned place preference in mice, even in the absence of further nicotine exposure. In the pre-experimental CPP optimization stage, mice in the saline control group showed no significant change in time spent in the white compartment, confirming that nicotine effectively induced place preference.

Recent studies indicated that l-THP, a dopamine receptor antagonist, was a promising therapeutic agent for addiction to psychostimulants and opioids, including cocaine, methamphetamine, and heroin (Jin et al., 1986; Mantsch et al., 2007; Xi et al., 2007; Xu et al., 2013). In this study, administration of 10 mg/kg l-THP alone significantly attenuated nicotine-induced CPP compared to the saline-treated nicotine control group.

LDN, an adjunctive therapy to methadone, could reduce relapse risk among opioid-addicted individuals (Parker et al., 2018; Rahn et al., 2011). The dose of LDN (0.3 mg/kg) was selected based on prior studies showing that LDN effectively modulates opioid signaling without precipitating a severe aversion (Bespalov et al., 1999; Parker and Rennie, 1992). In our experiment, administration of 0.3 mg/kg LDN alone did not significantly alter nicotine-induced CPP. Gerrits et al. reported that repeated administration of 3 mg/kg naltrexone diminished cocaine relapse in rats by modulating the endogenous opioid system, particularly through changes in β-endorphin levels (Gerrits et al., 2005). Increasing the dose of naltrexone also increase its side effects, compromising patient’s tolerance and compliance (Schmitz et al., 2014; Schmitz et al., 2009). The combination of 10 mg/kg l-THP and 0.3 mg/kg LDN markedly attenuated the established nicotine-induced CPP. The observation that mice in the l-THP and l-THP + LDN groups spent more time in the saline-paired chamber could be a pattern consistent with the development of aversion. However, several lines of evidence did not support aversion being the explanation. First, after successful CPP establishment, nicotine reward increased the time that mice spent in the white (nicotine-paired) compartment, despite their innate preference for the dark compartment. Following drug treatment, this duration decreased relative to the post-nicotine stage but did not fall below the baseline level measured before CPP acquisition. This pattern indicates that the pharmacological interventions attenuated the nicotine-induced reward effect without inducing additional aversion to the previously drug-paired environment. The doses of l-THP and LDN used were selected from literature demonstrating that they are neutral in rodent CPP paradigms, such that they do not induce preference or aversion at the chosen concentrations (Bespalov et al., 1999; Parker and Rennie, 1992; Su et al., 2013). Secondly, the extensive clinical use of l-THP, with acceptable patient compliance, provides a real-world context that pronounced intrinsic aversion is not an effect of this drug at therapeutic doses (Bespalov et al., 1999; Liu et al., 2005; Parker and Rennie, 1992; Su et al., 2013). The doses of l-THP and LDN used in this study have been reported to be behaviorally neutral in CPP paradigms when administered alone, such that they do not induce conditioned aversion. Preference scores returned toward baseline levels rather than shifting below baseline which would be indicative of aversive conditioning. The observed decrease in CPP is unlikely due to a nonspecific aversive effect of the treatments themselves, but rather an attenuation of nicotine-induced reward.

Mice in the 0.3 mg/kg LDN group and in the 10 mg/kg l-THP plus 0.3 mg/kg LDN combination group showed significantly elevated plasma β-endorphin levels compared to both the nicotine group and the 10 mg/kg l-THP group. This elevation could be a compensatory response to opioid receptor blockade rather than a direct enhancement of reward signaling. Given that LDN acts as an opioid receptor antagonist, it may trigger a homeostatic feedback mechanism that promotes the secretion of endogenous opioid peptides, including β-endorphin, in an attempt to restore receptor activity. Because the receptors are partially blocked, the released β-endorphin cannot effectively activate the μ-opioid receptor–mediated pathway (Corder et al., 2013; Nikolarakis et al., 1987; Spanagel et al., 1991; Zalewska-Kaszubska et al., 2008). Under such conditions, a state of “invalidated opioid signaling” may emerge, in which biochemical indicators of reward (β-endorphin elevation) are dissociated from the behavioral experience of reward (blocked CPP). The increase in β-endorphin is likely a compensatory response to sustained receptor blockade rather than an enhancement of reward signaling. It is noted that the relationship observed between elevated plasma β-endorphin levels and reduced nicotine preference in the present study is correlational. While the biochemical findings are consistent with our proposed mechanistic hypothesis, a direct causal relationship between β-endorphin changes and the observed behavioral effects has not been established. Accordingly, the proposed compensatory opioid mechanism should be interpreted as a plausible hypothesis rather than a definitive causal explanation.

In summary, several limitations in the present study are identified. First, nicotine reward was assessed using a single behavioral model, which captured motivational effects and did not cover all aspects of nicotine dependence. Second, the study was conducted exclusively in male mice, and potential sex-dependent effects were not evaluated. Third, the observed association between plasma β-endorphin levels and behavioral outcomes was correlational and did not establish causality. The collective evidence nonetheless supports the conclusion that the combination of the dopamine receptor antagonist l-THP and the opioid receptor antagonist LDN was more effective than l-THP alone in attenuating nicotine-induced reward. The combination of l-THP and LDN exerted its effect by modulating dopaminergic signaling pathways and endogenous opioid systems. The dual actions synergistically reduced nicotine-induced CPP, resulting in a greater attenuation of nicotine reward compared with single-agent treatments. Furthermore, the combined administration of LDN and l-THP was associated with a compensatory elevation in β-endorphin levels, consistent with known responses to opioid receptor blockade and an adaptive feedback process instead of an enhanced reward signaling. This interpretation is consistent with a working mechanistic hypothesis previously presented (Simmons and Self, 2009; Spanagel et al., 1991).

Future studies may further examine the efficacy of this combination in other nicotine-related behavioral paradigms, such as self-administration or relapse models, as well as explore underlying neurochemical changes in key reward-related brain regions. From a translational perspective, the observed synergistic effect may support future clinical studies of combination strategies aimed at improving efficacy while minimizing side effects.

## Data Availability

The original contributions presented in the study are included in the article/Supplementary Material, further inquiries can be directed to the corresponding authors.
