# Supplementary material for: Synergistic effects of levo-tetrahydropalmatine and low-dose naltrexone on nicotine conditioned place preference in mice: a dual-target strategy based on dopamine and opioid systems
Source: Front Pharmacol. 2026 Jan 14;17:1697258. doi: 10.3389/fphar.2026.1697258 (PMC12847241; doi:10.3389/fphar.2026.1697258)
Supplement: Supplementary file 1 [file Supplementaryfile1.docx]

Supplemental Materials for

**Synergistic Effects of Levo-Tetrahydropalmatine and Low-Dose Naltrexone on Nicotine Conditioned Place Preference in Mice: A Dual-Target Strategy Based on Dopamine and Opioid Systems**

Kun Feng^1,2#^, Yiran Zhao^1#^, Lu Liu^2^, Shan Kang^2^, Mingming Yu^1^, Sherwin K. B. Sy^3^, Zhihua Lv^1*^, Meixing Yan^2*^

**1** School of Medicine and Pharmacy, Ocean University of China, Qingdao 266003, PR China

**2** Women and Children Hospital, Qingdao University, Qingdao 266034, China

**3** Department of Statistics, State University of Maringá, Maringá, Paraná 87020-900, Brazil

# These authors contributed equally to this work and are co-first authors.

*Correspondence: lvzhihua@ouc.edu.cn (Zhihua Lv); Meixing Yan (meixing@163.com)

**Table S1. Conditions to optimize conditioned place preference model prior to drug testing**

| **No.** | **Adaptation Training**  **(Days)** | **Route of Administration** | **Training Duration (Days)** | **Nicotine Dose (mg/kg)** | **Training Time per Session (min)** |
| --- | --- | --- | --- | --- | --- |
| 1 | 1 | Intraperitoneal Injection | 6 | 1.0 | 45 |
| 2 | 0 | Intraperitoneal Injection | 3 | 1.0 | 30 |
| 3 | 0 | Intraperitoneal Injection | 3 | 2.0 | 30 |
| 4 | 3 | Subcutaneous Injection | 6 | 0.5 | 30 |
| 5 | 0 | Subcutaneous Injection | 3 | 0.3 | 20 |
| 6 | 1 | Subcutaneous Injection | 4 | 0.8 | 60 |
| 7 | 0 | Subcutaneous Injection | 3 | 0.5 | 30 |
| 8 | 0 | Subcutaneous Injection | 3 | 0.3 | 20 |

**Animal Species:** Kunming mice and C57BL/6 mice were primarily selected for the experiments.


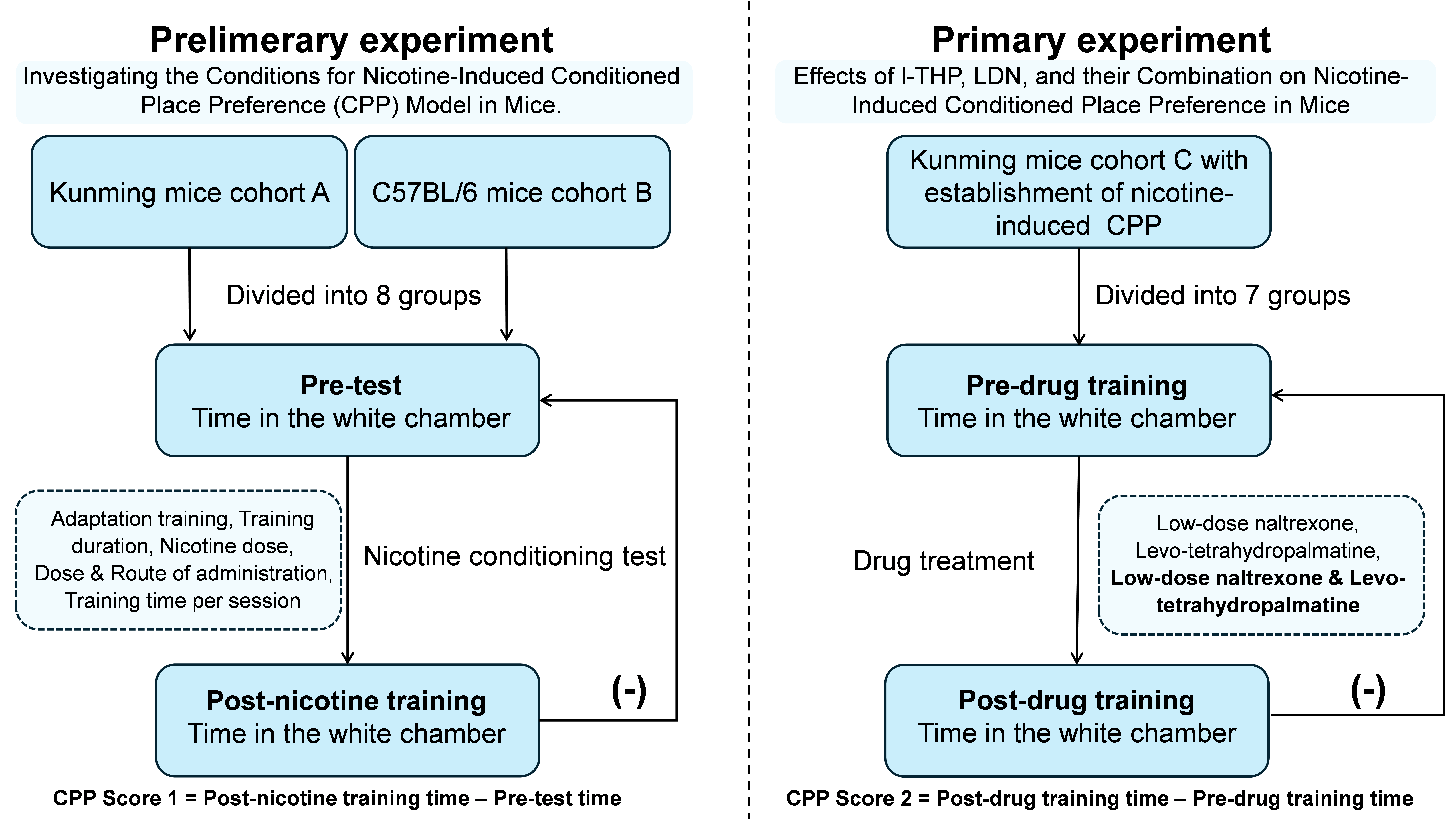


**Fig. S1.** Overview of study cohorts and CPP scoring across all experimental phases.


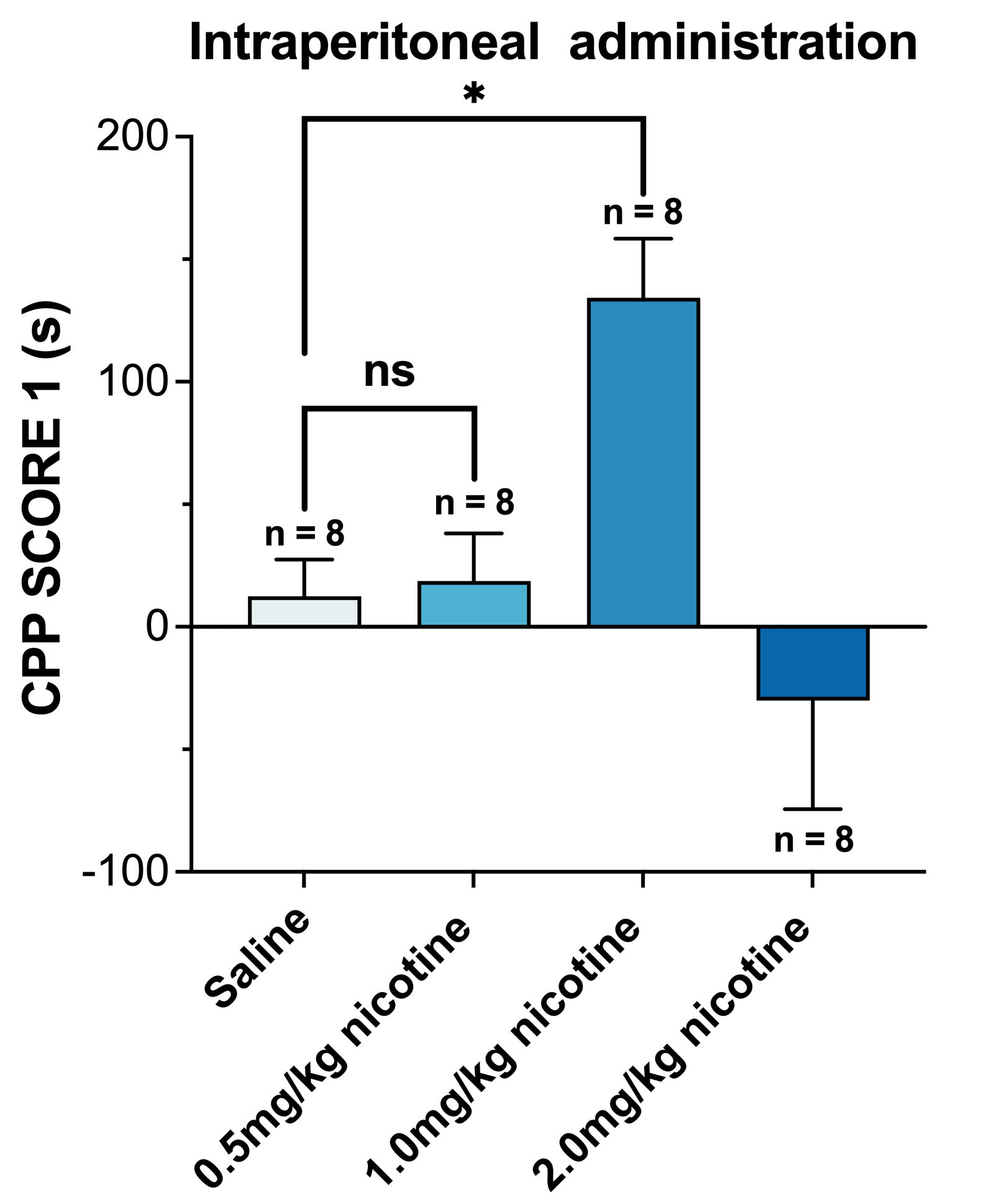


**Fig. S2.** Effect of intraperitoneal administration of nicotine doses on conditioned place preference (n = 8 per group, mean ± SEM). The animals received nicotine for 5 days by intraperitoneal (i.p.) administration of 0.5, 1.0, and 2.0 mg/kg nicotine. CPP SCORE 1 reflects the nicotine-induced change in white compartment preference (calculated as post-nicotine training time minus pre-test time). Statistical analyses were performed using one-way analysis of variance followed by post-hoc Tukey’s HSD test. *P < 0.05.


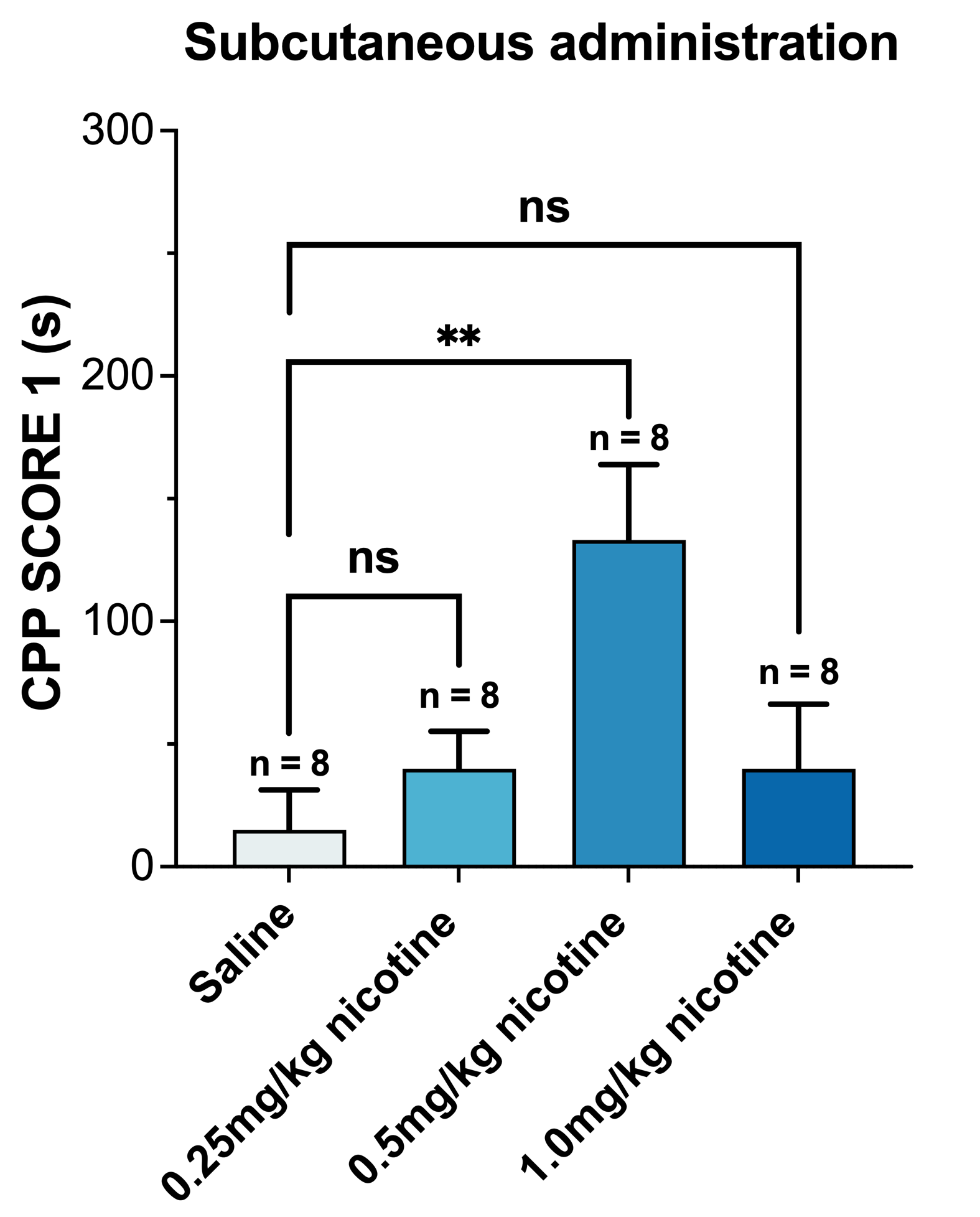


**Fig. S3.** Effect of subcutaneous administration of nicotine doses on conditioned place preference (n = 8 per group, Mean ± SEM). The animal received subcutaneous nicotine for 5 days at doses of 0.25, 0.5, and 1.0 mg/kg nicotine. CPP SCORE 1 reflects the nicotine-induced change in white compartment preference (calculated as post-nicotine training time minus pre-test time). Statistical analyses were performed using one-way analysis of variance followed by post-hoc Tukey’s HSD test. **P < 0.01.
